# Primary healthcare system readiness for the prevention and management of non-communicable diseases in Nepal

**DOI:** 10.21203/rs.3.rs-9699438/v1

**Published:** 2026-07-15

**Authors:** Chandani Singh Nakarmi, Sanju Bhattarai, Elizabeth C. Rhodes, Meghnath Dhimal, Phanindra Prasad Baral, Bikram Poudel, Binuka Kulung Rai, Anupama Bishwokarma, Sushmita Mali, Asmita Adhikari, Aarati Dhakal, Alina Bharati, Sangita Manandhar, Surakshya KC, Sashi Silwal, Bikram Adhikari, Soniya Shrestha, Felix Teufel, Dinesh Timalsena, Yunika Acharya, Donna Spiegelman, Archana Shrestha

**Affiliations:** 1 Research and Development Division, Dhulikhel Hospital-Kathmandu University Hospital, Dhulikhel, Kavre, Nepal; 2 Center for Methods in Implementation and Prevention Science, Yale School of Public Health, New Haven, Connecticut, USA; 3 Hubert Department of Global Health, Rollins School of Public Health, Emory University, Atlanta, Georgia, USA; 4 Nepal Health Research Council, Ramshahpath, Kathmandu, Nepal; 5 Epidemiology and Disease Control Division (EDCD), Nepal, Ministry of Health and Population, Teku, Kathmandu, Nepal; 6 Department of Biostatistics, Yale School of Public Health, Yale University, New Haven, Connecticut, USA; 7 Department of Public Health, Kathmandu University School of Medical Sciences, Dhulikhel, Kavre, Nepal; 8 Institute of Implementation Science and Health, Kathmandu, Nepal

**Keywords:** Non-communicable diseases, Primary healthcare system, Service readiness, PEN program

## Abstract

**Background:**

In 2016, Nepal adopted the World Health Organization’s Package of Essential Non-Communicable Disease Interventions (PEN) to address the rising burden of non-communicable diseases (NCDs). This study evaluated the primary healthcare system's readiness to prevent and manage major NCDs, including cardiovascular diseases (CVDs), diabetes mellitus (DM), and chronic respiratory diseases (CRDs), and associated factors.

**Methodology:**

We conducted a convergent parallel mixed-methods study between 2020 and 2022. We assessed 105 primary healthcare facilities selected through multistage stratified random sampling. Data were collected using the WHO Service Availability and Readiness Assessment (WHO-SARA) tool and analyzed using survey-weighted descriptive and multivariable linear regression. Simultaneously, we conducted qualitative interviews with 23 health authorities and 47 health service providers involved in PEN implementation. Interview transcripts were thematically analyzed using WHO-SARA domains.

**Results:**

NCD service readiness was highest for CVDs (48.4, 95% CI: 44.5–52.3), followed by DM (40.8, 95% CI: 36.1–45.5), and CRDs (34.8, 95% CI: 30.8–38.9). Primary Healthcare Centers (PHCCs), primary healthcare facilities in hilly regions, or those charging user fees, demonstrated higher NCD service readiness than health posts, mountain-region primary healthcare facilities, and those without user fees. Qualitative findings associated higher NCD service readiness with better infrastructure, medical supplies, training opportunities, and social health insurance. High staff turnover and limited medical supplies hindered NCD service delivery, particularly in health posts and remote regions.

**Conclusion:**

Primary healthcare facilities in Nepal lack equipment, medicines, trained staff, and NCD guidelines. The government could enhance NCD service readiness by building capacity and improving the availability of medical supplies.

## Introduction

Non-communicable diseases (NCDs) account for 71% (41 million) of all global deaths, and three-fourths (77%) of these deaths occur in low- and middle-income countries (LMICs) [1]. Nearly 80% of the NCD morbidities in the world are from one of the four major NCDs: cardiovascular diseases (CVDs), chronic respiratory diseases (CRDs), diabetes mellitus (DM), and cancers [2]. The probability of dying between the ages of 30 and 70 years from one of these NCDs is 17.8% globally and 21.5% in Nepal [3]. The overall prevalence of NCDs among Nepalese individuals aged 15–69 years is 55.4%. The burden of hypertension (27.3%) [4], type-2 DM (8.5%) [5], and chronic obstructive pulmonary disease (COPD) (11.7%) [6] is substantial. NCDs in Nepal contribute to 61.2% of disability-adjusted life years (DALYs) in Nepal [7] and poses a significant burden on the health system of Nepal, which is primarily financed by out-of-pocket payments [8,9]. More than 50% of total out-of-pocket health expenditures are allocated to NCDs [10].

In response to the large and increasing burden of NCDs, the government of Nepal implemented the National Multisectoral Action Plan (2014–2020) [11] and later endorsed the WHO’s Package for Essential Non-Communicable Disease (hereafter referred to as the PEN program) in 2016 [12]. The WHO PEN program provides an equitable framework for initiating primary care in low-resource settings, such as Nepal [13]. In the context of Nepal, PEN sets a minimum standard for the prevention and management of major NCDs (hypertension, diabetes, asthma, COPD, and breast and cervical cancer). The PEN program implementation encompasses strengthening national capacity and scaling up NCD care through primary healthcare settings. [12]. In 2017, the government of Nepal piloted the PEN program in two districts (Illam and Kailali) and gradually expanded it to 31 of the 77 districts in 2018 and 2019 [12,14]. A feasibility study in two PEN-piloted districts highlighted several gaps in health system preparedness for PEN program initiation, namely insufficient training of health workers, lack of medical supplies, and limited diagnostics services, and underscored the need to strengthen the healthcare delivery system before scaling up the PEN program nationally [14].

In LMICs such as Nepal, the primary healthcare system is the preferred approach for delivering basic health services, including those for NCDs [15]. A well-functioning primary healthcare system is critical, particularly when NCDs require extended or even lifelong healthcare support, from early risk identification and NCD case identification to management [16,17]. However, studies in Nepal have reported overall NCD service readiness across primary, secondary, and tertiary healthcare facilities, yet no study has comprehensively evaluated the primary healthcare system's readiness to manage NCDs [9,18]. This knowledge gap can thwart the government’s efforts to achieve Sustainable Development Goal 3.4, i.e., reducing premature mortality from NCDs by one-third by 2030 [19]. Our study aims to evaluate the primary healthcare system's readiness for the prevention and management of CVDs, DM, and CRDs, and explore factors associated with readiness for each NCD-specific service. The study's findings will help the government and policymakers develop strategies to improve the readiness of primary healthcare facilities, which is crucial for the successful implementation and scaling up of PEN program activities in Nepal and other low- and middle-income countries (LMICs) with similar health system contexts.

## Methodology

### Study design

**Figure 1:**
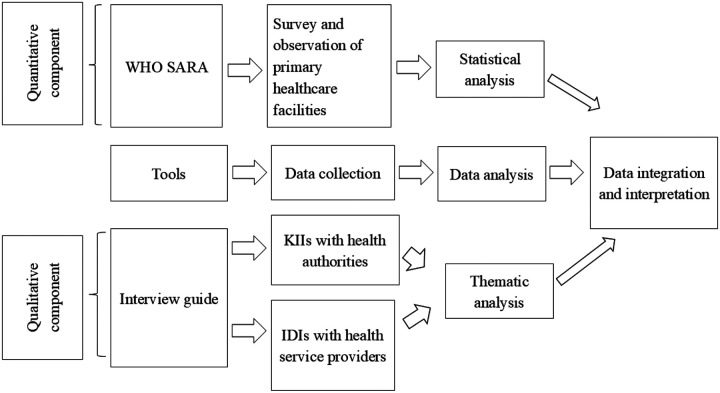
Convergent parallel mixed methods study design WHO SARA: World Health Organization Service Availability and Readiness Assessment, IDI: In-Depth Interviews, KII: Key Informant Interviews

We conducted a convergent parallel mixed-methods study to evaluate the primary health system's readiness to implement the PEN program in Nepal. The qualitative and quantitative data were collected in parallel, analyzed separately, and then merged for interpretation [20]. We quantitatively assessed the readiness of primary healthcare facilities for the screening and management of three major NCDs: diabetes mellitus (DM), cardiovascular diseases (CVDs), and chronic respiratory diseases (CRDs) [21]. Additionally, we conducted qualitative interviews with health authorities and health service providers involved in the PEN program implementation (Figure 1).

### Study setting

Nepal is a lower-middle-income country in Southeast Asia with an area of 147,516 km^2^ [22] and a total population of 29,112,480 [23]. Geographically, Nepal's landmarks are spread across three ecological zones: the Mountains, the Hills, and the Terai [24]. Administratively, it is divided into 7 provinces, within which lie 77 districts, each subdivided into rural and urban municipalities. All health-related activities are implemented under the close supervision of three tiers of government: federal, 7 provincial, and 753 local governments [9,25]. The federal and provincial governments oversee secondary and tertiary healthcare, while local governments are responsible for primary healthcare. The primary-level healthcare is provided through a network of 189 Primary Health Care Centers (PHCCs) and 3,794 health posts [26]. The PEN program in Nepal encompasses capacity building for primary healthcare providers through structured 4-day PEN training and the delivery of NCD services by trained providers in primary healthcare facilities, including health posts and PHCCs. District- and provincial-level secondary hospitals, along with tertiary-level specialized hospitals, serve as referral points for managing NCD-related emergencies and complications [27].

## Quantitative component

### Sampling

We calculated the minimum required sample size to estimate the NCD service readiness in primary healthcare facilities using the one-sample mean formula, n=Zα22×σ2d2. Assuming a 95% confidence level, a standard deviation (σ) of 17.6 units based on the 2015 Nepal health facility survey mean diagnostic index for diabetes services for public facilities [28], and a margin of error of ± 3.5 units, the minimum required sample size is 97 [29]. After adding an 8% non-response, the calculated sample size was 105.

**Figure 2:**
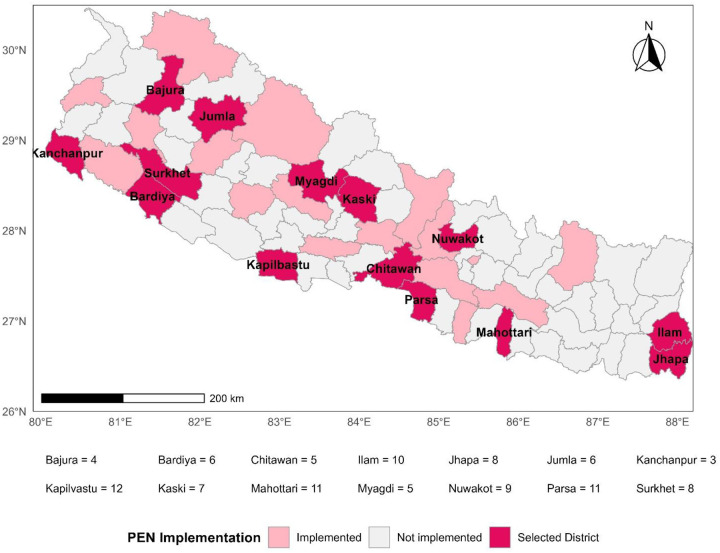
Geographic distribution of sampled primary healthcare facilities across 31 PEN-implemented districts of Nepal

We applied a multistage stratified random sampling to select primary healthcare facilities from the sampling frame of 31 districts implementing PEN as of 2018–2019, distributed across all 7 provinces. In the first stage, we randomly selected 14 PEN-implemented districts by sampling two districts from each province using STATA software [30]. In the second stage, all primary healthcare facilities from the sampled districts (653 health posts and 41 Primary Health Care Centers, or PHCCs) were listed and used as the sampling frame. We stratified primary healthcare facilities by rural and urban areas, randomly selecting PHCCs and health posts in proportion to their numbers within each stratum. PHCCs were oversampled as they represented only 5.9% of the total primary healthcare facilities in the sampled districts. The final survey sample comprised 71 health posts and 34 PHCCs, totaling 105 primary healthcare facilities (Figure 2).

### Data collection tools and techniques

We collected data between August 2020 and April 2022. We adapted the World Health Organization’s Service Availability and Readiness Assessment (WHO-SARA) tool, following the guidelines outlined by the national PEN manual of Nepal, to assess the readiness of primary healthcare facilities to deliver NCD-related services [21]. The WHO-SARA tool has been previously used in Nepal and several countries to assess the NCD-specific service readiness [9,31–34]. We used the WHO-SARA health facility inventory tool to collect information on the availability of NCD-specific equipment, drugs, and guidelines, and a health service provider questionnaire to obtain information related to PEN training, user fees, and monitoring activities by the higher government. To collect data, eight trained research assistants contacted primary healthcare facilities in charge via telephone to schedule on-site visits at their convenience. The research assistants explained the purpose of the visit, obtained written informed consent, and conducted face-to-face interviews using a paper-based questionnaire. They also verified service providers’ responses by directly observing facilities for equipment, drugs, and guidelines.

## Measurement of variables

### Outcome variables

We assessed three outcome variables in this study: the readiness of primary healthcare facilities to prevent and manage diabetes mellitus (DM), cardiovascular diseases (CVDs), and chronic respiratory diseases (CRDs). We calculated a mean composite NCD-specific service readiness score based on the availability of tracer indicators identified in the World Health Organization's SARA reference manual. The selected SARA indicators were also verified with the national PEN manual and Nepal's national essential drug list [35]. Based on the WHO-SARA manual, we grouped tracer items into three domains: i) staff training and guidelines, ii) equipment and diagnostics, and iii) essential medicines. We ascertained readiness in each domain described in Table 1. The value “1” was assigned if tracer items were present, and “0” was assigned if items were unavailable in primary healthcare facilities. The mean readiness score for each domain was calculated by summing the item values and then dividing the sum by the number of items, followed by multiplication by 100 (Supplementary Table 1). The overall NCD-specific service readiness ranged from 0 to 100. A higher readiness score indicated better service readiness for managing non-communicable diseases (NCDs).

### Explanatory variables

We ascertained the association between NCD-specific service readiness and the explanatory variables: type of primary healthcare facility (PHCCs/health posts), area (rural/urban), region (mountain/hills/lowlands), and the applicability of a separate user fee for NCD services (yes/no). The explanatory variables were selected based on the literature review [9,31,36]. Research assistants interviewed health service providers to obtain information about user fees and monitoring and supervision visits for the PEN program. Health service providers’ responses were further verified with charge sheets and registers, if available.

### Data management and analysis

We checked for missing entries on the same day that the data were collected. We resolved the problem of missing entries through a follow-up visit or phone call with the health facility in charge. Data were entered into online forms created in the Kobo Toolbox [37], exported into Excel, and imported to R version 4.3.2 [38] for cleaning and analysis.

During the analysis, the sample statistics were weighted to account for unequal selection probabilities and reflect a nationally representative sample of primary healthcare facilities. The sampling weight, which is the inverse of the selection probability for sample units, was based on stratification. First, district weights were calculated as the inverse of the selection probabilities for PEN-implemented districts in each province. Second, the weight for primary healthcare facilities was calculated based on their stratification by primary healthcare facilities’ setting (rural/urban) and type (health post/PHCC). Finally, the overall sampling weight was calculated as the product of two intermediate weights: the district weight and the primary healthcare facility weight. Categorical variables were summarized with frequencies and percentages, along with 95% confidence intervals, after applying sampling weights. NCD service-specific readiness scores for diabetes, CVDs, and CRDs were reported as means with 95% confidence intervals. We calculated weighted mean NCD service readiness scores by primary healthcare facility type and presented them with confidence intervals. We performed survey-weighted multivariable linear regression using the “svyglm” function from the survey package in R to identify factors associated with NCD service readiness. The “svyglm” function accounted for the complex survey design by applying survey weights, accounting for clustering, and ensuring robust variance estimation. Results were reported as regression coefficients (β) with 95% confidence intervals (CIs). Statistical significance was set at p-value < 0.05.

## Qualitative methods

### Sampling

We purposively sampled health authorities involved in planning, implementing, and monitoring the PEN program, as well as health service providers who delivered PEN services at the primary healthcare level. We conducted 23 key informant interviews (KIIs) with health authorities (Supplementary Table 2) and 47 in-depth interviews (IDIs) with health service providers (Supplementary Table 3) who had received the PEN program training. The sample size estimation for IDIs and KIIs was determined by code and meaning saturation [39]. The research team regularly conducted debriefing sessions to discuss data collection and saturation. We continued data collection until no new themes emerged in the qualitative data analysis.

### Data collection

We developed semi-structured interview guides for key informant interviews (KIIs) with health authorities and in-depth interviews (IDIs) with health service providers following the WHO-SARA manual. The interview guides covered four main aspects of the WHO-SARA manual: human resources, guidelines, equipment, and medicines for NCD management. The guides were initially developed in English, then translated into Nepali, pilot-tested with two health service providers and one health authority from non-participating health institutions and refined. More than half of the interviews were conducted in person (n = 41); the remaining interviews were conducted virtually via telephone (n = 10) or online using Google Meet (n = 19) due to COVID-19-related travel restrictions and social distancing requirements. The interviews were conducted by a research coordinator (CSN), who holds a master’s degree in public health, and two research officers (SM and SS), who hold master’s degrees in sociology, as well as two research assistants (AD and AB), who hold bachelor’s degrees in public health. All interviews were conducted in a private space within primary healthcare facilities or health offices and recorded on a digital audio recorder. The average interview duration was 54 minutes (range: 23–77 minutes) for health authorities and 41 minutes (range: 22–56 minutes) for health service providers. Following each day of data collection, the research team discussed emerging interview data and revised interview guides as needed through an iterative process.

### Data management and analysis

Research assistants transcribed all IDIs and KIIs verbatim in Nepali, and transcripts were compared against their respective audio files for accuracy and de-identified. We employed an inductive-deductive approach to thematic analysis [36,37], initially identifying themes from the qualitative data and subsequently organizing them according to the WHO SARA domains [21]. The research team reviewed one-third of the transcripts in each category to identify data issues and developed separate codebooks for health authorities and health service providers. The codebooks included a list of codes, their definitions, and examples of code application, and were developed through an iterative process with inputs from an experienced qualitative researcher (ER). The Dedoose software (version 7.0.23) [40] was used to manage and code textual data. Initially, two coders independently coded three or more interviews in each category and then matched their results to calculate percentage agreement. Any disagreements in code application were resolved through subsequent discussions, followed by iterative revisions to the codebook. After obtaining a minimum of 80% agreement, the coders independently coded the remaining interviews. The coders searched, coded, and categorized excerpts in the dataset, identifying recurring themes and discussing all emerging codes and meaningful patterns throughout the coding process. The research team conducted consecutive discussions to identify important emerging themes and mapped them to the WHO SARA framework [21].

### Data integration

We integrated quantitative and qualitative findings during the interpretation phase to identify the areas of convergence, divergence, and complementarity. We applied a contiguous narrative approach, presenting qualitative and quantitative findings in separate sections of the results. The first half of the results section reports findings from quantitative primary health facility assessments, NCD service readiness, and associated factors, and the second half presents key findings from qualitative research, offering a contextual explanation of the NCD readiness domains and associated factors [41]. The research team simultaneously reviewed quantitative analyses and emerging themes from qualitative analyses to assess the convergence, divergence, and complementarity of the findings. Both quantitative and qualitative data were given equal priority during the study.

## Results

### Background characteristics and operational aspects of primary healthcare facilities in Nepal

Table 2 presents unweighted and weighted distributions of primary healthcare facility characteristics; all descriptions are based on weighted estimates to ensure national representativeness. Of the total 105 primary healthcare facilities, 94.1% were health posts. In terms of geographical distribution, more than half of the primary healthcare facilities were in rural areas (57.1%) and from the lowlands (52.4%). Nearly one-third (35.6%) of primary healthcare facilities offered 24-hour emergency services, typically operating for a median of 7 hours (Q_1_:7, Q_3_:7). A few (6.8%) primary healthcare facilities implemented social health insurance, and 29.7% charged separate user fees for some NCD services. The median distance to the nearest referral center was approximately 16 kilometers (Q_1_:9, Q_3_:25). Most (84.8%) of the primary healthcare facilities reported no monitoring and supervision visits for the PEN program (Table 2).

### Availability of basic diagnostic equipment, medicines, trained staff, and guidelines for the management of NCDs

**Figure 3:**
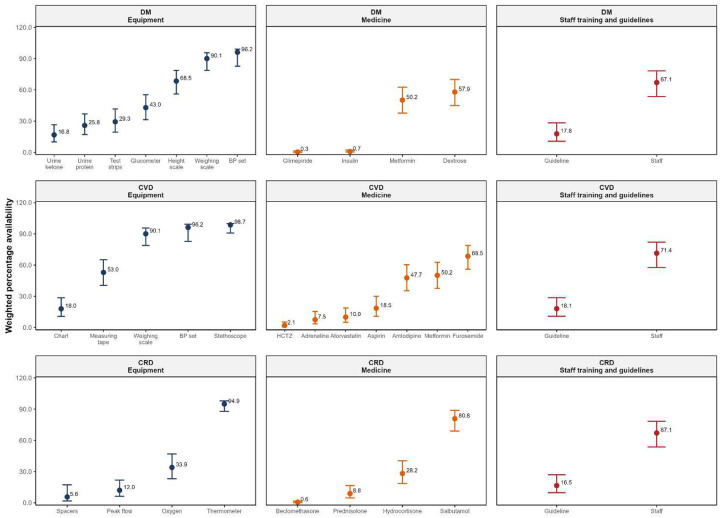
Weighted percentage availability of basic diagnostic equipment, medicines, guidelines, and at least one trained staff for the management of DM, CVDs, and CRDs in primary healthcare facilities (n=105) DM: Diabetes Mellitus, CVDs: Cardiovascular Diseases, CRDs: Chronic Respiratory Diseases

Most primary healthcare facilities had weighing scales (90.1%, 95% CI: 78.8–95.7%), blood pressure measuring apparatus (96.2%, 95% CI: 82.8–99.2%), and stethoscopes (98.7%, 95% CI: 90.8–99.8%). Glucometers, blood glucose test strips, and urine ketone dipsticks, which are used to diagnose diabetes, were available in 43% (95% CI: 31.4–55.3%), 29.3% (95% CI: 19.5–41.6%), and 16.8% (95% CI: 10.1–26.5%) of primary healthcare facilities, respectively. Peak flow meters and inhaler spacers were available in only 12% (95% CI: 6.3–21.8%) and 5.6% (95% CI: 1.7–17.3%) of primary healthcare facilities, respectively. Nearly half of primary healthcare facilities had metformin (50.2%, 95% CI: 37.7–62.6%) and amlodipine (47.7%, 95% CI: 35.4–60.3%) available. Atorvastatin was available in only 10% (95% CI: 5.1–18.8%) of primary healthcare facilities, whereas salbutamol tablets were available in 80% (95% CI: 69.0–88.9%). The availability of trained staff (71.4%, 95% CI: 57.7–82.0%) and guidelines (18.1%, 95% CI: 10.9–28.6%) was highest for CVDs compared to CRDs and DM. Two-thirds (67.1%, 95% CI: 53.6–78.2%) of primary healthcare facilities had at least one trained health service provider for the management of DM and CRDs (Figure 3).

### Readiness of the primary healthcare facilities for the management of NCDs

**Figure 4:**
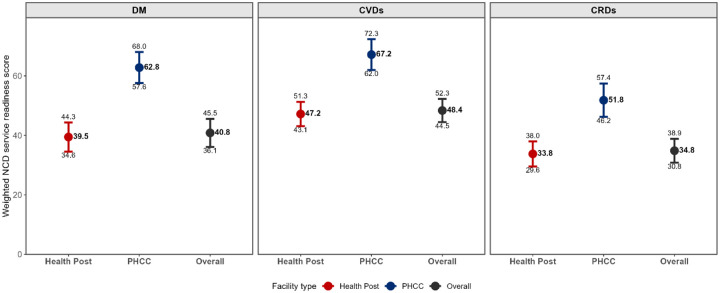
Readiness scores of primary healthcare facilities for the management of DM, CVDs, and CRDs (n=105) CVDs: Cardiovascular diseases, DM: Diabetes Mellitus, CRDs: Chronic Respiratory Diseases

Figure 4 illustrates NCD-specific service readiness across health posts, PHCCs, and overall primary healthcare facilities. The mean service readiness score for primary healthcare facilities was highest for CVDs at 48.4 (95% CI: 44.5–52.3), followed by DM at 40.8 (95% CI: 36.1–45.5), and CRDs at 34.8 (95% CI: 30.8–38.9). Overall, health posts had low service readiness for all NCDs: CVDs at 47.2 (95% CI: 43.1–51.3), DM at 39.5 (95% CI: 34.6– 44.3), and CRDs at 33.8 (95% CI: 29.6–38.0). Conversely, PHCCs, compared to health posts, demonstrated higher service readiness scores for CVDs (67.2, 95% CI: 62.0–72.3), DM (62.8, 95% CI: 57.6–68.0), and CRDs (51.8, 95% CI: 46.2–57.4).

### Factors associated with primary healthcare facilities' readiness for the management of DM, CVD, and CRDs

In multivariable linear regression analysis, PHCCs demonstrated higher service readiness scores compared to health posts for CVDs (β = 9.88, 95% CI: 0.37 to 19.34), DM (β = 11.90, 95% CI: 0.37 to 23.43), and CRDs (β = 13.18, 95% CI: 4.96 to 21.40). Compared to primary healthcare facilities in mountain regions, those in hills had higher readiness for CVDs (β = 17.63, 95% CI: 10.18 to 25.08) and CRDs (β = 16.43, 95% CI: −13.74 to 3.20). Likewise, the primary healthcare facilities with the provision of separate fees for some components of NCD services had higher readiness compared to those without user fees for CVDs (β=11.75, 95% CI:5.05 to 18.44), DM (β=14.21, 95% CI: 5.44 to 22.98), and CRDs (β=6.79, 95% CI: 0.74 to 12.84) (Table 3).

### Qualitative findings

We mapped the qualitative findings to WHO SARA domains and presented the key findings under three overarching themes: i) staff training and guidelines, ii) equipment and diagnostics, and iii) basic medicines (Table 4). The qualitative findings examined the readiness of primary healthcare facilities across the SARA domains and identified key discrepancies in NCD-specific service readiness by primary health facility type, region, and user-fee application. The qualitative findings added contextual depth to the quantitative findings, reinforcing the overall results.

#### Staff training and guidelines

##### Availability of PEN-trained staff

None of the primary healthcare facilities had staff solely dedicated to the PEN program. Many health service providers reported having 1 to 3 PEN-trained staff in their primary healthcare facilities, yet they managed multiple programs, including maternal and child health, family planning, and communicable diseases. All participants unanimously identified staff adjustment following federalization as the principal reason for the shortage and inequitable distribution of PEN-trained staff in primary healthcare facilities and health offices.

“After federalization, there was an exodus of permanent staff to the northern region. Only half of the sanctioned positions in primary healthcare facilities are fulfilled.”

-Public Health Inspector (HA2), Lowlands, District level

Most participants felt overburdened by staff shortages, and the addition of the PEN program further increased their responsibilities, compromising NCD service delivery. Some primary healthcare facilities and health offices temporarily hired untrained staff to offset staffing shortages but were unable to continue NCD services. Replacing vacant positions was particularly challenging for remote primary healthcare facilities; even after hiring new staff, staff retention remained uncertain.

“Temporary staff usually leave after a year of service, and we again have to recruit someone to fill in the vacant positions.”

-Public Health Section Officer (HA18), Hill, Provincial level

While the shortage of PEN-trained staff was ubiquitous, few primary healthcare facilities, mostly PHCCs, in urban lowlands and hilly regions mentioned having adequate staff. One PHCC in the lowlands was even being upgraded to a district hospital and was overstaffed. Despite having adequate and competent health service providers, many PHCCs were overburdened by high patient volumes and unable to conduct detailed patient examinations. Yet the practice of team-based care and task sharing was less common in both PHCCs and health posts.

“Team-based care and task sharing are not widely practiced. Teamwork and task sharing will ensure the provision of NCD services in primary healthcare facilities, even in the absence of PEN-trained workers.”

-HA18, PEN focal person, Province, Hill

In PHCCs, medical officers examined patients with NCDs and prescribed medicines, while paramedics provided counseling. Some PEN-trained medical officers and paramedics also reported organizing coaching sessions for untrained staff. In a few instances, PEN orientations were also organized for community leaders and female community health volunteers. Some participants believed that medical officers in PHCCs were competent and could provide NCD services based on peer coaching. Health service providers in health posts, in contrast, were less receptive to PEN orientation and peer mentoring than those in PHCCs and preferred waiting for PEN training to be provided.

“Not all health service providers in our health facility are trained, but they can manage NCD patients properly and provide NCD services as I have oriented them about the PEN program.”

-Medical officer (HSP5), PHCC, Lowlands

Overall, PEN training enhanced health service providers’ capacity to deliver NCD services, including diet education, blood pressure assessments, and the referral of emergency cases. However, some participants noted disparities in participant selection, with the PEN training being more focused on medical officers and paramedics. Nurses, who were not primarily involved in the examination and management of patients with NCDs, were less prioritized for PEN training. Even PEN-trained nurses were not providing NCD services due to other duties and obligations.

“Although our nurses are PEN-trained, they are assigned responsibilities of birthing, antenatal care, and family planning; therefore, other paramedics look after NCD patients in outpatient departments.”

-Public Health Inspector (HSP19), Health post, Hill

##### Availability of NCD guidelines

Many PEN-trained health service providers mentioned receiving a manual during the training session, which they refer to estimate CVD risk, perform clinical examinations, prescribe medications, and refer patients. Occasionally, some health service providers didn’t receive hard copies of the PEN manuals, and a few misplaced or lost them after a natural disaster demolished the primary healthcare facilities’ infrastructure.

“Previously, we had the PEN manual, but a landslide wrecked the health facility, and we lost the only guideline that we had.”

-Senior AHW (HSP13), Health post, Hill

Health service providers often cited the unavailability of a CVD risk estimation chart as the reason they did not estimate CVD risk in patients. A few proactive health workers with access to computers and printers printed digital copies of CVD risk estimation charts and used them to calculate CVD risk among patients; however, these printed copies could be easily misplaced.

“Handouts can get misplaced. If we could have provided separate risk prediction charts like flip charts, it would have been much easier for health service providers.”

-Public Health Officer (HA4), District level, Lowlands

Some health service providers occasionally consulted the PEN manual, particularly when patient flow was low in health facilities. Having low patient volume gave them time to consult NCD guidelines, and a few of them mentioned keeping them in the patient examination room so they could easily refer to them when they had questions or needed guidance in clinical decision making.

“I have a manual provided during the training session, which I follow for patients’ diagnoses.”

-Medical officer (HSP9), PHCC, Hill

The availability of the PEN manual was inconsistent across primary healthcare facilities, though some urban primary healthcare facilities reported adequate training resources.

“We have good training resources, including the PEN manual. We have been providing the services following the guidelines and utilizing the resources we have.”

Public Health Inspector (HSP19), Health post, Hill

##### Equipment and diagnostics

Many health service providers cited the availability of equipment, such as weighing scales, measuring tapes, blood pressure measurement sets, and stadiometers, in their primary healthcare facilities, while a few reported a lack of these basic instruments. Equipment, such as glucometers provided during initial training, was not maintained, and consumables, including glucose test strips and urine protein strips, were not supplied, or only expired strips were made available. Several health service providers reported having nonfunctional glucometers for blood glucose measurement. The unavailability of functional glucometers largely disrupted NCD services at health posts, as they lacked laboratory analyzers.

“During my incumbency at this PHCC, neither glucometers nor blood glucose test strips were provided. From what I understand, they were once supplied, and there hasn’t been any maintenance, restocking, or follow-up since then; obviously, they didn’t last indefinitely.”

-Medical Officer (HSP9), PHCC, Hill

Few health service providers cited the increased availability of glucometers and blood glucose test strips from senior citizen programs for adults aged 70 years and older. Some primary healthcare facilities even charged minimal user fees to NCD patients for specific diagnostic and therapeutic procedures. This helped primary healthcare facilities maintain the availability of NCD services without running out of essential equipment.

“We charge a nominal fee for blood glucose and lipid profile tests. This fee helps us maintain a consistent supply of reagents and kits to keep the lab operational.”

- Medical officer (HSP2), PHCC, Lowlands

In terms of medical supplies, PHCCs were better equipped than health posts. Few health service providers acknowledged the availability of sophisticated equipment, including electrocardiogram (EKG/ECG) machines, laboratory analyzers, and X-ray diagnostic equipment. Although PHCCs had more advanced equipment than health posts, peak flow meters were unavailable for CRD diagnosis, prompting many participants to demand them. Some health authorities reported distributing peak flow meters to the PEN trainees, but many either misplaced them or did not use them due to high operating costs.

“A metered dose inhaler (MDI) and Drug Powdered Inhaler (DPI) are required to operate a peak flow meter, which costs nearly 450 to 550 Nepalese rupees, and it is challenging to manage these health care costs.”

-WHO PEN field coordinator (HA5), central level

In the absence of peak flow meters, healthcare providers relied on history-taking and clinical examination with stethoscopes, and those in PHCCs could also use X-rays to diagnose CRDs. The lack of diagnostic equipment in health posts and Primary Health Care Centers (PHCCs) led health service providers to resort to history-taking, vital signs, and physical examination to diagnose NCDs. The routine lack of functional diagnostic equipment in primary healthcare facilities compelled patients with NCDs to forgo testing and seek services at higher centers.

“We have not been able to provide lab and diagnostic services to patients with NCDs due to the unavailability of essential medicines; therefore, patient flow has decreased.”

-Health inspector (HSP46), Health post, Terai

##### Essential medicines

Many participants reiterated the unavailability of essential medicines as a critical bottleneck for NCD service delivery. The primary healthcare facilities mostly provided essential medicines, including amlodipine, hydrochlorothiazide, atenolol, metformin, atorvastatin, and salbutamol tablets free of cost. However, the supply of these NCD drugs, particularly atorvastatin, was mostly irregular, discouraging NCD patients from continuing to seek services.

“When basic medicines like amlodipine and metformin are not available in the health post, patients visiting our health post get discouraged and directly visit higher centers for medicine refills.”

-Health Assistant (HSP25), Health post, Mountain

Many health service providers reported their ordeal with prescribing medicine, as not all medicines specified in the PEN protocols were covered in the national essential drug list. For instance, the PEN protocol recommends nebulization and administration of steroids and salbutamol via metered dose inhalers (MDIs) or drug-powdered inhalers (DPIs), but these drugs were not freely available. Health service providers, particularly health posts, were required to continue using phased-out medications (e.g., salbutamol tablets) or to refer CRD patients to higher centers. The situation was dire in remote, mountainous regions, where NCD patients had limited access to other healthcare institutions.

“Since we don’t have inhalers and nebulizers, we immediately refer patients experiencing chronic obstructive pulmonary disease (COPD) and asthma exacerbations to higher centers. We only have salbutamol tablets and must prescribe them despite side effects”

-Medical officer (HSP9), PHCC, Hill

Health service providers in PHCCs had increased availability of NCD medicines due to social health insurance schemes, which provided them with greater prescribing flexibility; however, not all NCD patients visiting PHCCs had health insurance. Some health service providers in PHCCs found it particularly challenging when some drugs for NCDs were available free of cost, while others were not. Patients without health insurance were either prescribed freely available essential drugs or asked to purchase non-essential medicines from private drug outlets, depending on their affordability and accessibility. There were also instances when PHCCs ran out of supplies, could not even offer NCD medicines through insurance, and faced patients’ criticism. Some health service providers also reported instances in which patients with NCDs discontinued services at primary healthcare facilities.

“When basic medicines like amlodipine and metformin are not available in our health post, patients are discouraged from revisiting our health post… If they need those medicines in the future, they will directly go to higher centers, thinking that the medicines will never be available in our facility.”

-Health Assistant (HSP25), Health post, Mountain

In addition to health insurance, the senior citizenship scheme ensured access to NCD medicines for older adults (>70 years) visiting primary healthcare facilities. NCD medicine procurement and supply chain management were also affected by federalism and the COVID-19 pandemic. Some participants reported that local municipalities increased the supply of NCD drugs after federalism, while many noted that federalization only complicated the procurement process for medicines. Due to COVID-19, primary healthcare facilities reported low stock of NCD drugs but increased supplies of thermometers, sanitizers, handwashing products, and masks for COVID-19 prevention and control.

“The province must purchase medicines in bulk, which complicates the procurement process. In every tendering process, the province faces one or another legal issue, and the tendering process gets delayed by two to three months.”

-Public Health Inspector (HA2), District level, Lowlands

In some remote primary healthcare facilities, the availability of NCD medicines did not always ensure patient accessibility due to topographical barriers, such as difficulty reaching these facilities during the rainy season, when transportation is not readily available. Some health service providers at adequately stocked primary healthcare facilities tactfully forestalled any potential drop-offs from treatment plans for patients with NCDs by providing refills during their facility visits, sufficient to sustain them when they are unable to access primary healthcare facilities.

“The roadblocks during the monsoon were also a major hindrance for patients. So, we provided adequate refills for about 2 months and counseled them to attend regular follow-ups at the nearest primary healthcare facility.”

-Health Assistant (HSP11), PHCC, Rural, Hill

### Integrated results

The quantitative and qualitative findings together provided a comprehensive understanding of Nepal’s primary healthcare system's readiness for NCD prevention and management.

Quantitative findings were supplemented by qualitative interviews, where participants attributed poor NCD service readiness to physical and systemic constraints, including inadequate infrastructure, topographical constraints, infrequent training opportunities, high staff turnover, and a lack of guidelines and supplies for NCD management. Additionally, federalization and the COVID-19 pandemic exacerbated existing disparities in NCD service readiness. Despite the challenges, some primary healthcare facilities with better infrastructure and capacity managed to provide NCD services through peer coaching, social health insurance schemes, application of minimal user fees for some NCD services, and local government support. These complementary findings provided a nuanced understanding of the systemic and contextual factors influencing the primary healthcare system’s readiness for NCD prevention and management.

## Discussion

We evaluated the readiness of Nepal’s primary healthcare system and associated factors for the prevention and management of DM, CVDs, and CRDs. The average NCD service readiness scores for all three NCDs were below the recommended threshold of 70 [42–44]; therefore, primary healthcare facilities, in general, were not optimally prepared to manage NCDs. NCD readiness scores varied by primary healthcare facility type, with higher readiness in PHCCs compared to health posts; geographical region, with higher readiness in the hills compared to the mountains; and applicability of separate user fees, with higher readiness in primary healthcare facilities that impose user fees. Qualitative findings provided context for the gaps in WHO SARA domains, showing how systemic gaps in infrastructure, human resources, equipment, and medicines led to uneven NCD service readiness. These system-level constraints were partially offset by social health insurance schemes in PHCCs, improving access to medications, human resources, and referrals. Beyond systemic factors, NCD service readiness was also influenced by broader contextual factors, such as COVID-19 and federalization, which complicated the procurement and allocation of NCD resources; yet some health offices and primary healthcare facilities defied these odds and continued to maintain NCD service delivery.

In our study, the overall service readiness scores for DM and CVDs were comparatively higher than those for CRDs (asthma and chronic obstructive pulmonary disease), a pattern also observed in studies from Bangladesh [33], Kenya [32], Myanmar [45], and a systematic review across multiple countries (sub-Saharan Africa, South Asia, Region of America, and European Region) [17]. The lower CRD readiness may be due to two reasons. First, DM and CVDs are often prioritized in global and national health strategies due to their high prevalence and the significant burden they impose on healthcare systems. Second, managing CRDs requires advanced equipment and specialized care (e.g., oxygen therapy and pulmonary rehabilitation) that may be difficult to set up and operate at the primary level. This finding, however, is concerning, as CRDs are one of the leading causes of mortality in Nepal [46,47].

Despite the variation in overall service-readiness scores, the availability of trained staff and guidelines was similar for DM, CVDs, and CRDs, possibly because the national PEN manual covers basic precepts for managing all three NCDs [12]. Some PHCCs had staff who were additionally trained to handle cardiac emergencies, including basic and advanced life support. Yet, one-third of primary healthcare facilities lacked PEN-trained personnel, consistent with studies from other resource-constrained settings, such as Bangladesh [34], India [48], and Vietnam [49]. The shortage of PEN-trained staff was widespread across Nepal's primary healthcare facilities, primarily due to staffing adjustments following the federalism reforms [50]. Prompt action by the government at all levels is required to track existing PEN-trained staff, train new healthcare staff, and deploy them prudently to ensure equitable distribution. Additionally, PEN-trained staff should take responsibility for orienting their colleagues through routine peer coaching to reinforce clinical competencies and ensure consistency in NCD service delivery. Furthermore, task shifting should be implemented by redistributing specific tasks, such as NCD screening, counseling, and follow-up, from highly trained health service providers (e.g., doctors) to mid-level providers (e.g., paramedics) [51,52].

Early NCD screening and management in primary health care settings relied on the availability of testing equipment; however, our study revealed wide variation in the availability of NCD-specific diagnostic equipment, with most primary health care settings lacking them. On the contrary, basic equipment, such as weighing scales, stethoscopes, and BP sets, were readily available in primary healthcare facilities, similar to studies in Nigeria [53], Pakistan [54], Tanzania [31], and Cambodia [55]. The widespread availability of basic equipment across countries likely reflects its utility beyond NCDs, given its relevance to the diagnosis and management of various health conditions, including NCDs. Consistent with a study in Pakistan [54], most primary healthcare facilities in our setting lacked functional equipment for diabetes management, possibly due to the high maintenance costs of glucometers and the non-reusable nature of lancets and testing strips. Thus, addressing these gaps in NCD-specific diagnostic equipment requires not only the provision of equipment but also sustainable maintenance strategies and consistent supply.

Only a few primary healthcare facilities in our study were equipped with peak flow meters for CRD diagnosis, similar to a study in Nigeria [53] and Tanzania [31]. Moreover, primary healthcare facilities lacked peak flow meters, spacers, and metered-dose inhalers (MDIs) to conduct peak flow tests, a recommended diagnostic test for CRDs in the PEN program. The unavailability of diagnostic equipment led health service providers to rely on history-taking and physical examination to diagnose and prescribe medications for patients with NCDs. This practice was commonplace in all primary healthcare facilities and could severely compromise the quality of care through inappropriate diagnosis and irrational use of drugs. The prolonged use of unwarranted medication can increase the risk of adverse drug events in patients with NCDs [56,57]; therefore, primary healthcare facilities should prioritize alternative objective diagnostic methods to improve diagnostic accuracy.

Our study revealed critical gaps in the readiness of primary healthcare facilities to provide essential NCD medicines, with this domain scoring the lowest among all assessed domains. Primary healthcare facilities, particularly health posts, were authorized to dispense limited NCD medicines [58], yet those basic medicines were also unavailable. Nearly half of the primary healthcare facilities lacked amlodipine and metformin, with the lowest availability for atorvastatin. The persistent shortage of NCD medicines in primary healthcare facilities could eventually lead to increased referrals to higher-level centers, overburdening them. Similar to studies in Tanzania [31] and Myanmar [45], primary healthcare facilities in our study lacked salbutamol and corticosteroid inhalers. As a result, primary healthcare facilities, primarily health posts, dispensed only salbutamol tablets despite their low effectiveness in CRD management. Patients with CRDs were compelled to buy medicines from private drug outlets, which are considerably expensive. In the long run, these regular out-of-pocket payments for NCD management could be catastrophic for poor patients living in rural and mountainous regions [59,60], given the chronic nature of NCDs. Consequently, many patients with NCDs drop out of the treatment plan and turn to unproven conventional treatment practices [61].

Compared with health posts, PHCCs in our study consistently had higher service readiness across all three NCDs. This difference may exist because PHCCs, in general, are more advanced than health posts in terms of infrastructure and resources, and serve as referral points for health posts [62]. Another possible reason for improved readiness could be the social insurance coverage in PHCCs [63]. Our qualitative findings also corroborate our reasoning, as several health service providers reported an increased supply of NCD medicines after liaison with the social health insurance board. Health posts, by contrast, lacked a social health insurance scheme and dispensed only a limited range of NCD drugs listed by the Ministry of Health and Population [64]. Hence, expanding social health insurance schemes to health posts could help initiate the PEN program at health posts, the first point of contact for patients with NCDs.

Our findings revealed regional disparities in NCD-specific service readiness, with higher readiness in the hills than in the mountains or the lowlands. This variability in service readiness could be due to differences in the timelines for PEN program initiation and scale-up across the sampled PEN implemented districts. As the PEN program was launched in 2016 in tandem with the social health insurance program in the Ilam, hills, and Kailali, lowland districts, these PEN-piloted districts received substantial support from the government and WHO for program initiation and continue to serve as model districts and hubs for PEN training and scale-up in hilly and lowland regions [65,66]. Primary healthcare facilities in the mountains, in contrast, are hard to reach [67], and the high altitude and rugged terrain typically impede the transport of medicines and equipment, and healthcare staff turnover is also high. Therefore, the government should develop and implement preemptive strategies to retain PEN-trained staff in the mountains and expedite the delivery of medical supplies to primary healthcare facilities.

Implementing small user fees for certain services (e.g., blood glucose testing and diabetic foot dressing) significantly improved NCD readiness in primary healthcare facilities in our study. Our findings further expand on Nepal’s national health facility survey results, which reported an average positive association between user fees and NCD readiness [68]. Despite this, given the cross-sectional nature of our data, we cannot conclude whether user fees causally lead to improved NCD service readiness or vice versa. User fees could enhance the autonomy of primary healthcare facilities to purchase medical supplies and cover additional operational costs; however, caution is required when implementing user fees, as they could deter low-income patients from accessing NCD services at these facilities [69].

## Policy implications

We propose three recommendations for all levels of government. **First,** the government should deploy PEN-trained personnel to primary healthcare facilities that experienced losses following federalism and plan training accordingly. For participants who are unable to attend in-person training, sessions should be live-streamed on alternative media platforms, and training materials should be organized into self-paced online modules. PEN trainers should provide supportive supervision to trainees and help them conduct peer coaching for their colleagues in primary healthcare facilities. Additionally, integrating PEN training components into the curricula of medical, nursing, and paramedic programs is essential for long-term sustainability. **Second,** the government should focus on building multi-sectoral partnerships, both within and outside the government, to support supply chain management. The national social health insurance program should be expanded to health posts, and other parallel programs (e.g., the senior citizenship program) should be integrated into the PEN program to scale up the program activities. **Third,** the government should allocate an adequate budget to print the PEN manual, CVD risk estimation charts, and other information, education, and communication (IEC) materials related to NCDs. The CVD risk estimation charts and NCD guidelines should be available as flip charts and posters to support health service providers during patient examinations. If hard copies are unavailable, at least soft copies of the CVD risk estimation charts and NCD guidelines should be circulated to health service providers.

## Strengths and Limitations

Our study has several strengths. First, this study is the first to comprehensively evaluate the readiness of primary healthcare facilities across Nepal's geographic regions. Second, trained research assistants collected data using the adapted WHO-SARA tool and verified the availability of essential items: NCD guidelines, equipment, and medicines. Third, the quantitative estimates were weighted to account for oversampling of PHCCs and for unequal selection probabilities of primary healthcare facilities resulting from the complex survey design. Finally, the qualitative findings support the quantitative results and reveal various contextual factors that contribute to higher or lower NCD service readiness.

Despite its strengths, our study has limitations. First, our study did not account for the design effect inherent in multistage sampling, which may have led to underestimation of standard errors and confidence intervals. Second, NCD service readiness was assessed based on the availability of tracer items: the mere presence of one functional piece of equipment or an unexpired drug was considered indicative of readiness, which overestimates NCD service readiness in understocked primary healthcare facilities. Third, information on the availability and monitoring of trained staff was obtained from the primary healthcare facility; however, in some cases, it could not be validated through record review. Therefore, health service providers may have underreported or overreported training and monitoring data due to social desirability bias, fear of repercussions, or an inability to recall accurate information. Thus, the possibility of information and social desirability bias remains in the study.

## Conclusion

The primary health system's readiness for services related to DM, CVDs, and CRDs reveals substantial deficits in human resources and in the availability of guidelines, equipment, and medications. The shortage of essential drugs was identified as a significant hindrance to the delivery of NCD services. High staff turnover was evident across all primary healthcare facilities, and this issue must be addressed to retain PEN-trained staff. The readiness of primary healthcare facilities for managing NCDs varied by topography, with minimal readiness in mountainous regions; by type of primary healthcare facility, with poor readiness in health posts; and by the applicability of user fees, with low readiness in facilities that did not impose user fees. Therefore, the government should consider these disparities in the primary healthcare system when allocating healthcare resources and scaling up PEN program activities. The federal, provincial, and local training centers should collaborate to identify primary healthcare facilities that lack personnel trained in PEN and to prioritize them for subsequent training sessions. Moreover, training centers should provide PEN manuals and CVD risk estimation charts to each participant attending PEN training sessions, enabling easy reference during clinical practice.

## Supplementary Material

This is a list of supplementary files associated with this preprint. Click to download.

- SupplementaryTable1.docx
- SupplementaryTable2.docx
- SupplementaryTable3.docx

## Figures and Tables

**Table 1:** Domains and tracer indicators assessing readiness for the management of DM, CVDs, and CRDs

Domains	Tracer indicators	Assessment
Staff training and guidelines	Guidelines for the diagnosis and management of DM, CVDs, and CRDs	Availability of the PEN manual or any standard treatment hard copy protocols for DM, CVDs, and CRDs in primary healthcare facilities.
	Staff trained in the diagnosis and management of DM, CVDs, and CRDs	Self-reported availability of at least one staff trained in PEN or completion of other relevant courses in diagnosis and management of DM, CVDs, and CRDs.
Equipment and diagnostics	DM: blood pressure (BP) apparatus, adult weighing scale, measuring tape or stadiometer, glucometer, blood glucose test strips, urine dipstick protein, and urine dipstick ketone	Availability of functioning digital or aneroid BP measuring apparatus, adult weighing scale, measuring tape or stadiometer, glucometer, blood glucose test strips, and valid urine strips for the measurement of protein and ketone.
	CVDs: BP measuring apparatus, adult weighing scale, stethoscope, measuring tape, and oxygen	Availability of functioning stethoscope, digital or aneroid BP measuring apparatus, adult weighing scale, measuring tape, oxygen, and CVD risk estimation charts.
	CRDs: stethoscope, peak flow meter, spacers for inhalers, and oxygen	Availability of functioning stethoscopes, peak flow meters, spacers for inhalers, and oxygen
Basic medicines	DM: metformin, sulfonylureas, insulin, dextrose injection	Availability of at least one valid metformin, sulfonylureas, insulin, and dextrose injection.
	CVD: calcium channel blocker, enalapril, furosemide, hydrochlorothiazide, atorvastatin, aspirin, and metformin	Availability of at least one valid calcium channel blocker, enalapril, furosemide, aspirin, hydrochlorothiazide, atorvastatin, and metformin
	CRDs: beclomethasone inhaler, salbutamol tablets, prednisolone, hydrocortisone injection, and epinephrine injection	Availability of at least one valid beclomethasone inhaler, salbutamol tablets, prednisolone, hydrocortisone injection, and epinephrine injection

DM: Diabetes Mellitus, CRDs: Chronic Respiratory Diseases, CVDs: Cardiovascular Diseases, PEN: Package of Essential Non-Communicable Diseases

**Table 2:** Background characteristics of primary healthcare facilities in districts where the PEN program was implemented (n=105)

Variable	Unweighted	Weighted
	n (%)	%
**Facility type**		
Health post	71 (67.6)	94.1
PHCC	34 (32.4)	5.9
**Area**		
Rural	52 (49.5)	57.1
Urban	53 (50.5)	42.9
**Ecological region**		
Mountain	10 (9.5)	6.2
Hills	39 (37.1)	41.4
Lowlands	56 (53.3)	52.4
**Opening hours, Median (IQR)**	7 (7.0, 10.4)	7 (7,7)
**Emergency service**		
Available	54 (51.4)	35.6
Not available	51(48.6)	64.4
**Health insurance**		
Implemented	30 (28.6)	6.8
Not Implemented	75 (71.4)	93.3
**User fee for NCD services**		
Yes	54 (51.4)	29.7
No	51 (48.6)	70.3
**Distance to nearest referral center (in kilometers)**	20 (11,30)	16 (9,25)
**Monitoring and supervision**		
In the last three months	2 (1.9)	1.3
More than three months ago	12 (11.4)	13.9
Never performed	91 (86.7)	84.8

n= frequency, IQR=Interquartile range (Q_1_, Q_3_), PHCC: Primary Healthcare Centers, IQR consists of the first quartile (Q_1_) and the third quartile (Q_3_)

**Table 3:** Factors associated with primary healthcare facility readiness for the management of NCDs (n=105)

Characteristics	CVD readiness		DM readiness		CRD readiness	
	Unadjusted β (95% CI)	Adjusted β (95% CI)	Unadjusted β (95% CI)	Adjusted β (95% CI)	Unadjusted β (95% CI)	Adjusted β (95% CI)
Facility type						
Health post	Ref	Ref	Ref	Ref	Ref	Ref
PHCC	16.52 (4.74 to 28.30) **	9.88 (0.37 to 19.34) *	21.42 (7.23 to 35.61) **	11.90 (0.37 to 23.43) **	14.63 (2.22 to 27.08) *	13.18 (4.96 to 21.40) **
Location						
Rural	Ref	Ref	Ref	Ref	Ref	Ref
Urban	−1.62 (−15.98 to 12.75)	1.12 (−5.44 to 7.68)	0.89 (−16.84 to 18.62)	3.76 (−7.21 to 14.72)	−3.69 (−19.87 to 12.49)	−0.37 (−8.16 to 7.42)
Region						
Mountain	Ref	Ref	Ref	Ref	Ref	Ref
Lowlands	−0.19 (−7.73 to 7.34)	−0.21 (−6.79 to 6.37)	−8.22 (−19.05 to 2.61)	−8.42 (−7.21 to 14.72)	−5.31 (−14.67 to 4.06)	−5.28 (9.36 to 23.50)
Hill	19.83 (13.54 to 26.13) **	17.63 (10.18 to 25.08) ***	12.84 (2.63 to 23.06) *	10.35 (−2.18 to 22.89)	17.76 (10.50 to 25.02) ***	16.43 (−13.74 to 3.20) ***
Separate fee						
No	Ref	Ref	Ref	Ref	Ref	Ref
Yes	17.38 (8.15 to 26.61) ***	11.75 (5.05 to 18.44) **	20.09 (8.13 to 32.05) **	14.21 (5.44 to 22.98) **	13.63 (3.02 to 24.23) *	6.79 (0.74 to 12.84) *

* p<0.05,

** p<0.01,

*** p<0.001

Ref: Reference category, DM: Diabetes Mellitus, HTN: Hypertension, CVDs: Cardiovascular Diseases. CRDs: Chronic Respiratory Disease

**Table 4:** Summary of qualitative findings mapped to the WHO Service Availability and Readiness Assessment (SARA) domains

WHO SARA domains	Key findings	Illustrative quotes	
		Health authorities	Health service providers
PEN-trained staff	Primary healthcare facilities lacked focal persons for NCDs.	*“NCD services are delivered by existing staff, without dedicated healthcare personnel for NCDs.”*	*“I look after 38 tuberculosis patients and 6 patients with leprosy. I feel overburdened and unsure where to focus.”*
	PEN training was uneven across the cadres.	*“PEN training was specifically prioritized for medical doctors and health assistants.”*	*“Out of 3 health service providers, 2 are nursing staff who haven’t received PEN training.”*
	PHCCs had more staff and were more adaptive to peer coaching.	*“Doctors in PHCCs can take leadership roles to train other staff and guide them.”*	*“Even in the absence of a PEN-trained provider, another will fill in to provide NCD services.”*
	High turnover of staff in remote primary healthcare facilities.	*“Post federalism, there was a pooling of senior healthcare professionals in urban areas.”*	*“Recently, 2 of our PEN-trained staff were transferred to another primary healthcare facility.”*
NCD guidelines	Hard copies of the PEN manual were not provided, lost, or misplaced.	*“We provided printed copies of the CVD risk estimation charts, but the handouts can be easily misplaced.”*	*“During the rainy season, our health post was flooded, and we lost the only PEN manual*.
	Health service providers with access to computers and printers used digital or printed copies of NCD guidelines.	*“Some committed health service providers printed and laminated CVD risk estimation charts at their own expense for future use.”*	*“The PEN training organizing team provided soft copies of the PEN manual, so we can access them on our laptops. We also took notes during the training.”*
Equipment and diagnostics	Non-functional equipment and the unavailability of consumables disrupted NCD service delivery.	*“Glucometers in primary healthcare facilities are not functional, and we lack sufficient test strips and blood pressure monitors.”*	*“Glucometers, weighing scales, stadiometers, and equipment are currently unavailable, and this has impeded NCD service delivery.”*
	PHCCs have a greater availability of NCD equipment than health posts.	*“Compared to health posts, PHCCs have better availability of essential equipment for NCD services.”*	*“Our PHCC has equipment like an ECG machine, X-ray machine, and blood analyzers. We offer blood sugar tests.”*
	Topographical barriers limited access to NCD diagnostic services.	*“Patients don’t visit primary healthcare facilities for routine tests, and the situation is worse during the rainy seasons, when vehicles are also unavailable.”*	*“Some patients must walk steep roads for two hours, while others must walk through landslide-prone areas.”*
Essential medicines	Frequent stockouts of freely available NCD medicines.	*“Health offices run out of stock when municipalities don’t purchase medicines.”*	*“Atorvastatin tablets were only supplied once and have been out of stock since then.”*
	Social health insurance and the Senior Citizen Program improved access to NCD medicines.	*“Due to the free availability of medicines through insurance schemes, NCD patients have started taking NCD drugs.”*	*“The senior citizenship scheme ensures the provision of NCD medicines for citizens (>70 years) with NCDs.”*
	Sustained NCD medicines shortages led to dropouts in remote areas.	*“High dropouts from NCD treatment plans in remote areas are due to the unavailability of NCD medicines and taboos related to medications.”*	*“When basic medicines like amlodipine and metformin are not available, patients are discouraged from revisiting our health* post.*”*

## Data Availability

The datasets used in this study are not publicly available due to the confidentiality of certain data.

## Abbreviations

CVDs: Cardiovascular diseases
DM: Diabetes Mellitus
CRDs: Chronic Respiratory Diseases
DALYs: Disability Adjusted Life Years
LMICs: Low- and Middle-Income Countries
NCDs: Non-communicable diseases
SARA: Service Availability Readiness Assessment
WHO-PEN: World Health Organization-Package of Essential Non-communicable Diseases
IEC: Information, Education, and Communication
HA: Health Authority
HSP: Health Service Provider
